# Insights Into Plant Surgery: An Overview of the Multiple Grafting Techniques for *Arabidopsis thaliana*

**DOI:** 10.3389/fpls.2020.613442

**Published:** 2020-12-10

**Authors:** Kai Bartusch, Charles W. Melnyk

**Affiliations:** ^1^Institute of Molecular Plant Biology, Department of Biology, ETH Zürich, Zurich, Switzerland; ^2^Department of Plant Biology, Swedish University of Agricultural Sciences and Linnean Center for Plant Biology, Uppsala, Sweden

**Keywords:** grafting, *Arabidopsis*, micrografting, organ transplantation, hypocotyl

## Abstract

Plant grafting, the ancient practice of cutting and joining different plants, is gaining popularity as an elegant way to generate chimeras that combine desirable traits. Grafting was originally developed in woody species, but the technique has evolved over the past century to now encompass a large number of herbaceous species. The use of plant grafting in science is accelerating in part due to the innovative techniques developed for the model plant *Arabidopsis thaliana*. Here, we review these developments and discuss the advantages and limitations associated with grafting various *Arabidopsis* tissues at diverse developmental stages.

## Introduction

**FIGURE 1:**
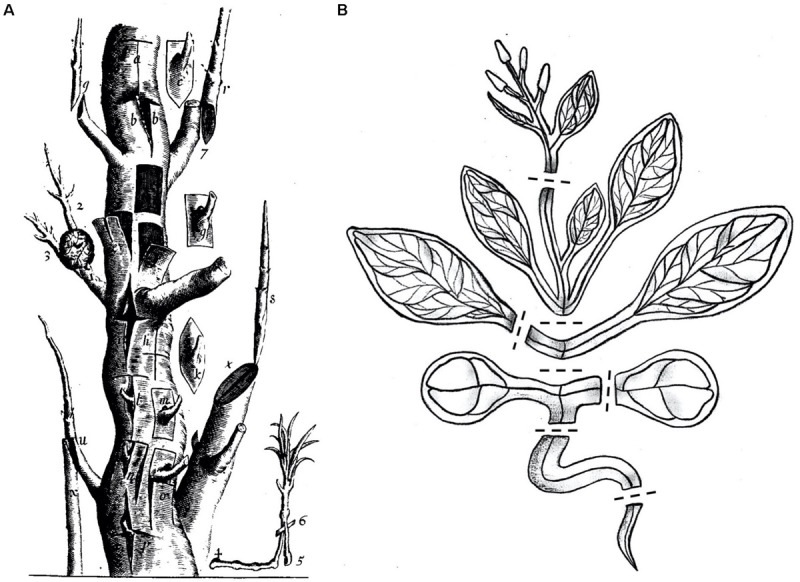
Multiple grafting techniques in woody and herbaceous plants. **(A)** In the 17th century there were already diverse grafting methods used which are illustrated in this exemplified trunk. Reprinted from Robert Sharrock’s “The history of the propagation and improvement of vegetables” (Sharrock, 1660). **(B)** *Arabidopsis thaliana*, a modern-day Sharrock’s tree. A cartoon showing *A. thaliana* cut at positions suitable for grafting including inflorescence stems, rosette stems, true leaf petioles, epicotyls, cotyledon petioles, hypocotyls, and roots. Dashed lines denote potential cutting patterns.

The transplantation of plant organs, commonly referred to as plant grafting, involves cutting and joining plant tissues from at least two different plants to generate a chimeric organism (Melnyk and Meyerowitz, 2015). Since ancient times grafting has been successfully applied for horticultural and agricultural purposes like improving plant vigor, enhancing disease and stress resistance, or for plant propagation. At first, grafting was established in woody plant species (Figure 1A) and more recently expanded to include diverse herbaceous plant species. By developing new graft combinations and automation techniques, over a billion plants are nowadays grafted worldwide, with an increasing number each year (Mudge et al., 2009; Lee et al., 2010).

In parallel, grafting of the model plant species *Arabidopsis thaliana* was established nearly 30 years ago and has developed extensively since (Figure 1B). However, rather than for propagation or stress tolerance, the initial motivation for *Arabidopsis* grafting was to study the long-distance movement of molecules. By grafting different *Arabidopsis* genotypes or even different species, such as a mutant and a wild-type whereby one genotype lacks the respective molecule or pathway, the appearance of a molecule not found in that genotype would signify mobility (Tsutsui and Notaguchi, 2017; Thomas and Frank, 2019). *Arabidopsis* grafting has led to major scientific discoveries which depend on long-distance transport of different molecules. For instance, the organ-to-organ transport of plant hormones (Matsumoto-Kitano et al., 2008; Ragni et al., 2011; Camut et al., 2019), RNAs (Brosnan et al., 2007; Molnar et al., 2010), proteins (Corbesier et al., 2007; Yoo et al., 2013; Takahashi et al., 2018), nutrients (Green and Rogers, 2004; Widiez et al., 2011), or secondary metabolites like glucosinolates (Andersen et al., 2013) have been already investigated. Notably, such studies were not often performed in traditionally grafted species such as apple or grape vine due to a lack of genetic resources and inadequate assaying techniques. Nowadays with whole genome sequencing and the development of RNA-Seq, sensitive hormone profiling, and quantitative mass spectrometry, these types of experiments can be performed on nearly any grafted plant species. Another relevant development is the use of *A. thaliana* as a tool to study the process of grafting itself (Yin et al., 2012; Melnyk et al., 2015). Due to its short generation time, extensive genetic resources, relatively small size and its great ability to graft to itself and to related species, *A. thaliana* is a powerful tool to study graft formation in detail. To date, significant process is being made in our understanding of how plants graft and the underlying mechanism (Goldschmidt, 2014; Melnyk, 2017c; Wang et al., 2017; Baron et al., 2019). We are beginning to understand processes involved such as wound healing and vascular differentiation (Melnyk et al., 2015, 2018; Matsuoka et al., 2016). In this context, the old enigma of graft failure (graft incompatibility) could be studied in greater detail in the near future by using a suitable incompatible grafting partner with *A. thaliana.* Using the genetic resources of *Arabidopsis* would allow communication, recognition, and tissue regeneration between incompatible grafted tissues to be examined in greater detail (Melnyk, 2017a). Moreover, using *Arabidopsis* to address the development and role of plasmodesmata formation across the graft junction could be studied further (Kollmann and Glockmann, 1985, 1991; Kollmann et al., 1985; Pina et al., 2012). *Arabidopsis* would also be highly relevant to study the mechanistic basis for rootstock-scion interactions such as stress tolerance and changes in plant vigor that are commonly observed in horticultural graft chimeras (Warschefsky et al., 2016). In contrast to these numerous advantages, it remains uncertain whether this enhanced *Arabidopsis*-based knowledge can be directly transferred to important horticultural species. However, is it likely that grafting shares common features in both woody and herbaceous plants and on some levels, including wound healing and vascular formation, there is likely to be some mechanistic conservation between grafting in different species (Goldschmidt, 2014; Melnyk, 2017c).

Grafting in *A. thaliana* was first reported for inflorescence stems (Tsukaya et al., 1993). Since this technique is limited to mature plants, another technique was developed nearly a decade later that involved grafting young *Arabidopsis* seedlings, termed micrografting (Turnbull et al., 2002). *Arabidopsis* micrografting has transformed our ability to graft – both due to the high success rates, rapid healing, fast numbers that can be done per hour and the large number that can be grafted per petri dish. Since this revolutionary technique has emerged, diverse grafting techniques have been described for hypocotyls (Turnbull et al., 2002; Marsch-Martínez et al., 2013; Melnyk, 2017b), epicotyls (Li et al., 2019), roots (Wang et al., 2011), and even for embryonic leaves (Yoo et al., 2013; Bartusch et al., 2020). Moreover, grafting techniques for older developmental stages have developed in parallel (Ayre and Turgeon, 2004; Chen et al., 2006; Nisar et al., 2012; Huang and Yu, 2015).

In this review, we discuss the various grafting techniques for the model plant species *A. thaliana* and present some of their respective advantages and limitations.

## Micrografting – the Transplantation of Seedling Tissues

**FIGURE 2:**
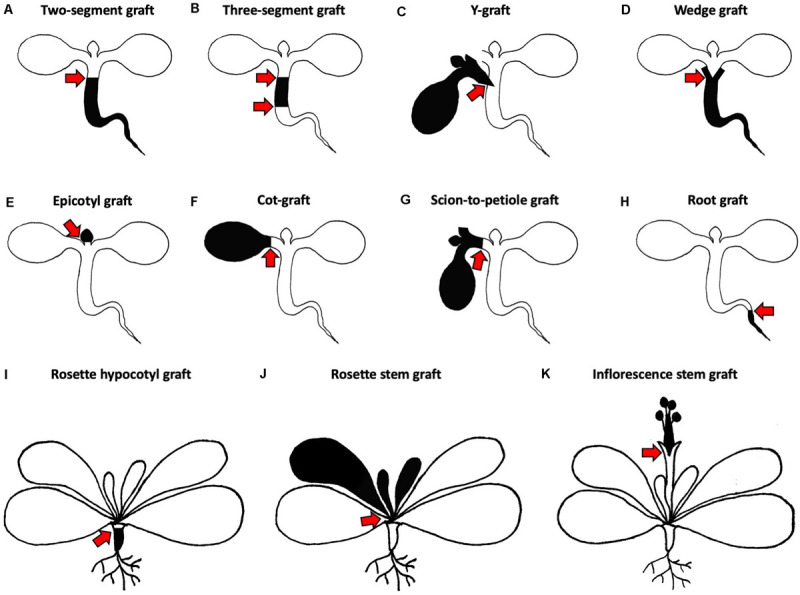
Different grafting methods for *Arabidopsis thaliana*. **(A–H)** Micrografting techniques in small plant seedlings. **(A)** A two-segment graft consists of a scion and rootstock joined via a horizontal cut in the hypocotyl region. **(B)** A three-segment graft combines an upper, middle and lower hypocotyl. **(C)** A Y-graft reconnects two scions and one rootstock in the hypocotyl region. **(D)** A wedge graft is a two-segment graft with a V-shaped cut scion inserted into a slit made in the rootstock. **(E)** An epicotyl graft maintains the cotyledons of the rootstock and the scion with true leaves is positioned on top. **(F)** A cot-graft unifies a donor cotyledon with a recipient plant. **(G)** A scion-to-petiole graft associates a donor scion and a recipient plant in the petiole region. **(H)** A root graft adds a donor root to a recipient plant. **(I–K)** Grafting techniques in older plants with mature rosettes. **(I)** A rosette hypocotyl graft consists of a mature scion rosette which is transplanted to the rootstock hypocotyl. **(J)** A rosette stem graft retains the lower leaves of the rootstock and transplants the upper part of the scion rosette. **(K)** An inflorescence stem graft is performed by joining the upper part of the scion inflorescence with the lower part of the rootstock inflorescence. Black and white colors indicate different origins of transplanted organs. Red arrows mark graft junctions.

The core idea of micrografting is to transplant tissues or organs at very early developmental stages (Figure 2; Turnbull et al., 2002). Although the handling of small seedlings needs practice and skill, it provides important benefits compared to the grafting of older and larger plants. Firstly, providing sufficient seedlings for grafting is not time or space-consuming because large numbers can be grown on media plates within several days. Moreover, the success of grafting can be assessed after only a few days and high throughputs of up to 80 plants per hour can be reached by an experienced grafter (Melnyk, 2017b). Additionally, seedlings show a great ability to regenerate since after grafting, the vasculature reconnects and growth resumes after a week (Yin et al., 2012; Melnyk et al., 2015). Diverse protocols were developed which describe suitable methods for different seedling’s tissues, each typically achieving high success rates of over 80% (Marsch-Martínez et al., 2013; Melnyk, 2017b; Bartusch et al., 2020). Apart from this, grafting of seedlings is not limited to *A. thaliana*. Other model species like *Eutrema salsugineum* (Li et al., 2019) and also important crop plants like *Brassica napus* (Ostendorp et al., 2016) and *Solanum lycopersicum* (Marsch-Martínez et al., 2013) were successfully grafted at seedling stage. However, micrografting typically needs further technical prerequisites like sterile conditions and a stereomicroscope (Turnbull et al., 2002). Depending on the method, special grafting tools are also necessary (Turnbull et al., 2002; Tsutsui et al., 2020). Nonetheless, the establishment of micrografting was transformative and the method is increasingly utilized in research and beyond. In the following, we focus on specific organs and tissues of *A. thaliana* that can be transplanted using the micrografting setup.

### Micrografting in the Hypocotyl Region

**FIGURE 3:**
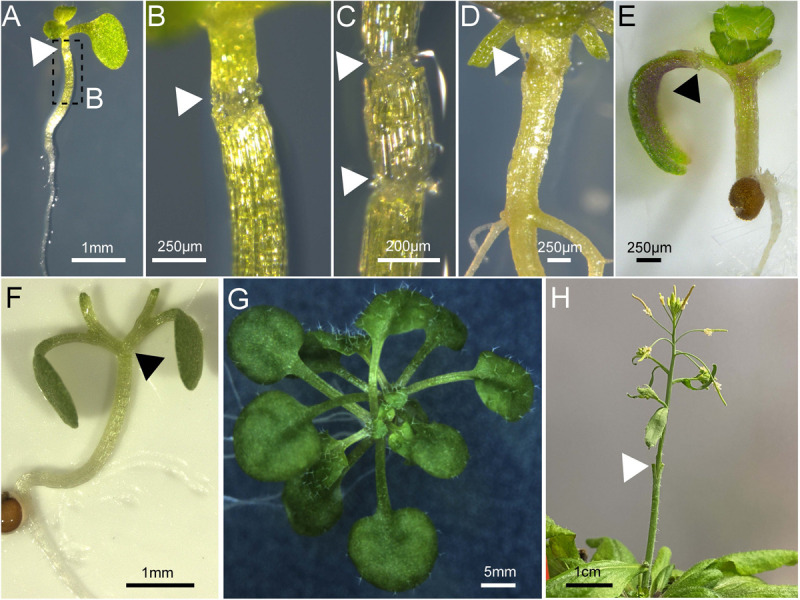
Various tissues grafted in *Arabidopsis thaliana*. **(A–D)** *Arabidopsis* hypocotyl micrografting performed on a 7-day old plant and imaged 10 days after grafting **(A–C)** or 48 days after grafting **(D)**. **(C)** Shows a three-segment graft. **(E)** *Arabidopsis* cotyledon micrografting performed on a 5-day old plant and imaged 7 days after grafting. **(F,G)** *Arabidopsis* Y-grafting performed on a 7-day old plant and imaged on day 0 **(F)** or 25 days after grafting **(G)**. An *Arabidopsis* inflorescence 4 days after grafting on a mature plant secured with a wedge graft. **(A–H)** Triangles (white or black) denote the graft junction and scale bars are indicated.

Traditionally, grafting aims to join a shoot, the so-called scion, and a rootstock in the stem region. *A. thaliana* is a characteristic rosette plant and its hypocotyl presents the most accessible stem-like tissue where a scion and rootstock can be fused. Thus, micrografting in the hypocotyl region of *A. thaliana* can provide an analogous tissue to stem grafting in woody or herbaceous horticultural plants (Turnbull et al., 2002). Still, the *Arabidopsis* hypocotyl is hard to access and visualization of the graft junction can be impaired, especially in older tissues (Figure 3D). Although the hypocotyl structure is similar to roots, it is thicker due to one extra cortex layer (Lin and Schiefelbein, 2001). In addition, the hypocotyl also contains chloroplasts which make it harder to see into the tissue and these also have a high autofluorescence signal.

Initially, two different techniques were developed for hypocotyl micrografting (Turnbull et al., 2002). The group of Ottoline Leyser developed a technique whereby hypocotyl micrografting was performed directly on the agar growth medium in 7–9 days old seedlings and grafts were assembled using a silicon tube across the graft junction to assist with attachment and stabilization (Turnbull et al., 2002; Bainbridge et al., 2014). The assembly of the graft is more time consuming but the tube seems to impair adventitious root growth (Turnbull et al., 2002). In parallel, the group of Colin Turnbull developed a technique whereby 4-day old seedlings were transferred to a membrane on top of wet filter papers prior to grafting so to provide a flat solid cutting surface. The cut scion and rootstock can be attached without a stabilizing tube which simplifies the procedure and saves time, but might promote adventitious root formation (Turnbull et al., 2002; Marsch-Martínez et al., 2013; Melnyk, 2017b). Sharp scalpels can be used for cutting (Turnbull et al., 2002; Marsch-Martínez et al., 2013). Alternatively, more expensive special surgical micro knives are recommended to increase surgical precision and improve grafting success rates (Andersen et al., 2014; Melnyk, 2017b; Bartusch et al., 2020). More recently, a micrografting chip was developed as a device to assist with *A. thaliana* grafting. This chip serves as growth, grafting and recovery facility in one. Thus, the grafting process can be facilitated compared to other techniques and makes it technically much less challenging (Tsutsui et al., 2020). In contrast to conventional hypocotyl micrografting success rates of up to 90 or 100% (Marsch-Martínez et al., 2013; Melnyk, 2017b), this chip method achieved a 24–88% success rate (Notaguchi et al., 2020; Tsutsui et al., 2020). The lower success rates observed with the chip could be due to agar surrounding the junction or the lack of manual adjustment of the graft junction.

Turnbull et al. (2002), introduced three different ways of joining scions and rootstocks in the hypocotyl region. The most popular approach is to combine one rootstock with one scion for the analysis of shoot-to-root or root-to-shoot molecule transport. The hypocotyl cuts are either performed horizontally or V-shaped to produce a horizontal two-segment graft (Figure 2A) or a wedge graft (Figure 2D), respectively. Horizontal cutting is easier and faster, but V-shaped cuts might promote attachment due to the greater surface area in contact between scion and rootstock. Today, the V-shaped cut is not commonly used and instead, most protocols use the horizontal cut (Figures 3A,B,D; Brosnan et al., 2007; Molnar et al., 2010; Melnyk, 2017b; Tsutsui et al., 2020). While Turnbull et al. (2002), left both cotyledons, more recent methods recommend removing one cotyledon so that the scion hypocotyl lies flat on the graft surface facilitating alignment between rootstock and scion (Brosnan et al., 2007; Melnyk, 2017b). It is also possible that removing one cotyledon partially reduces auxin levels which might otherwise promote adventitious root formation (Steffens and Rasmussen, 2016). The removal of both cotyledons was also tested, but here, a supplement of sucrose is needed to reach high success rates (Marsch-Martínez et al., 2013). In contrast, sucrose addition appears to diminish grafting success when one cotyledon is left. In this case, sucrose probably promotes adventitious root formation rather than graft formation (Melnyk, 2017b).

To test shoot-to-shoot transport of molecules, two-shoot grafting, referred to as Y-grafting (Figures 2C, 3F,G), was developed by inserting a V-shaped cut scion into a slit made on the hypocotyl of the recipient plant (Turnbull et al., 2002). Later on, a three segment graft was developed where the hypocotyl is cut twice and three pieces – an upper, middle, and lower hypocotyl – are rejoined (Figures 2B, 3C; Melnyk, 2017b). Such three segment grafts conceptually resemble inter-stock grafts seen in horticulture (Melnyk and Meyerowitz, 2015). As other approaches of joining plant organs in the hypocotyl regions already exist in other plant species, it is very likely that further modifications will appear in the future. For instance, an inverted Y-grafting method was used in *Lotus japonicus* by inserting a V-shaped cut rootstock into the recipient plant hypocotyl to investigate root-to-root signaling (Magori et al., 2009). This inverted Y-graft could probably be also implemented in *A. thaliana*. As micrografting in the hypocotyl region can only focus on shoot-root signaling, micrografting was also extended to other seedling’s tissues (Wang et al., 2011; Yoo et al., 2013). Nevertheless, the general micrografting setup developed by Turnbull et al. (2002), is still widely used, with some minor modifications, due to its high success rates (>80%), rapid speed (40–80 grafts per hour) and ease with which many grafts can be grown in a small space. However, specialist tools are beneficial, and training is required to master the technique (Table 1). In our experience, the greatest challenges are avoiding damaging the seedlings and not adding too much water to the grafting plates (Melnyk, 2017b). There are also possible limitations with assaying movement of molecules since there appears to be bias in the direction of mobility for some signals. For instance, studies with small RNA movement observed rapid movement from shoot to root but much slower movement of an RNA silencing signal from root to shoot (Brosnan et al., 2007; Molnar et al., 2010; Melnyk et al., 2011) that seemed to progress through a cell-to-cell, non-vascular pathway (Liang et al., 2012). This greater efficiency from shoot to root may represent the directionality of the phloem (source to sink movement) and should be kept in mind when designing movement experiments with hypocotyl grafting.

**TABLE 1:** The main *Arabidopsis* grafting techniques.

**Tissue**	**Age of plants**	**Advantages**	**Disadvantages**	**Most appropriate to study**
Hypocotyl (Turnbull et al., 2002; Marsch-Martínez et al., 2013; Melnyk, 2017b)	5–7 days old	Fast (40–80 per hour). Y-grafts and three segment grafts possible. Grafted seedlings take up little space. Widely used.	Technically challenging to learn. Specialized equipment facilitates grafting.	Graft junction formation. Shoot to root movement. Y-grafts for shoot to shoot movement.
Stem (Huang and Yu, 2015)	10 days to 6 weeks old	Grafts form before the inflorescence emerges.	Technically challenging to learn. Modest amount of growth space required.	Shoot to shoot movement. Shoot to root movement. Shoot to inflorescence movement.
Inflorescence (Nisar et al., 2012)	1–2 months old	Fast. Easy to perform. Little specialist equipment required.	Modest amount of growth space required. Grafts form late in development.	Shoot to inflorescence movement. Graft junction formation.
Cotyledon (Bartusch et al., 2020)	4–7 days old	Fast (40 per hour). Grafted seedlings take up little space.	Technically challenging to learn. Cotyledons are soon replaced by true leaves.	Leaf to shoot movement. Leaf to root movement.
Root (Wang et al., 2011)	6 days old	Root junction potentially easier to visualize than hypocotyl junction.	High lateral root production above the junction is likely. Success rates unknown.	Root to root movement. Shoot to root movement. Graft junction formation.

*Tissues, plant ages, and relevance are indicated.*

### Micrografting in the Petiole Region

Micrografting of embryonic leaves, the cotyledons, was also reported in *A. thaliana* where one cotyledon of the donor plant is transferred to a cut petiole of a recipient plant, a technique we abbreviate as cot-grafting (Figures 2F, 3E; Yoo et al., 2013; Bartusch et al., 2020). Cotyledons show an incredible regenerative capacity despite their relatively short life span of a few weeks. The vasculature between both organs connected as fast or even faster than in hypocotyls (Bartusch et al., 2020). The first published cot-grafting protocol performed cotyledon surgery directly on agar medium and reached a low success rate of <2% (Yoo et al., 2013), whereas a flat solid cutting surface and an elevated temperature level was recently identified to be critical for reaching success rates up to 92% (Bartusch et al., 2020). Cot-grafting was previously used to investigate cotyledon-shoot interactions (Yoo et al., 2013; Bartusch et al., 2020). In the future, cot-grafting could be used to study cotyledon-root signaling as well. Still, the cotyledon as an embryonic leaf does not represent a true leaf and is most relevant to study early plant development. However, the removal or substitution of cotyledons does affect plant development and biomass production (Wang et al., 2019; Bartusch et al., 2020). Techniques to graft true leaves in the petiole regions have been developed but are not yet published (Akira Iwase and Keiko Sugimoto, RIKEN, Japan). Such techniques would provide a new approach to study leaf-leaf, leaf-shoot, and leaf-root interactions. Moreover, we recently described a new approach for joining organs where scions are transferred to cut petioles, termed scion-to-petiole micrografting (Figure 2G). In this case, one cotyledon from each scion is cut-off before joining (Bartusch et al., 2020). Shoot-shoot interactions can be studied similar to Y-grafts, although it remains unknown whether this technique can be used to study the mobility of molecules.

To conclude, micrografting in the petiole region is probably less relevant for studying graft formation and more interesting for long distance signaling research. Still, cotyledon-derived substances could affect graft formation in hypocotyls and cot-grafting can provide deeper insights in these processes. A natural extension of this process will be the implementation of true leaf grafting.

### Micrografting in the Root Region

Plants can form connections between their roots, a process termed natural root grafting, which was already described in the 19th century (Reum, 1835; Goeppert, 1842). Root grafting is widespread across many species, especially trees. Grafts can form within a plant or between different plants of the same species but distantly related species forming root grafts is very uncommon (Graham and Bormann, 1966). These vascular connections between trees can be efficiently used for spreading pathogens. Thus, root grafting has to be considered in forest management and in forest plantations (Epstein, 1978). Despite its abundance in nature, nothing is known about its underlying mechanism. These natural grafts presumably form through contact and fusion which differ from human-derived grafts that normally involve cutting or deep wounds. *A. thaliana* micrografting in the root region could contribute to our understanding of root grafting. The *Arabidopsis* root is easy to access and to visualize compared to the hypocotyl, and many respective mutant and well described reporter lines for the root are already available. However, artificial micrografting in the root region often induces adventitious root formation rather than graft formation (Turnbull et al., 2002). Despite these challenges a root micrografting technique was developed (Figure 2H) where horizontal cuts and graft junction formation occur just below the root-shoot junction (Wang et al., 2011). Still, root micrografting appears challenging and likely has low success rates. To simulate natural root grafting where roots fuse in the absence of wounding, induction conditions need to be determined like the identification of suitable developmental stages and environmental parameters. To our knowledge, there are no reports about natural root grafting in *A. thaliana* so far, except inter-species root connections caused by parasitic plant infections such as by *Phtheirospermum japonicum* (Ishida et al., 2016). If *A. thaliana* is not capable of natural root grafting, the establishment of another model system might be necessary such as the use of a woody species. Better understanding natural grafting could contribute to our understanding of graft evolution and also help better manage pathogen transfer in natural environments.

## Transplanting Organs of Mature Plants

While micrografting usually needs sterile conditions and special tools, grafting of *A. thaliana* at later developmental stages does not need sterile conditions and the plants are grown on soil (Ayre and Turgeon, 2004; Huang and Yu, 2015) or in hydroponic conditions before grafting (Chen et al., 2006). Additionally, widely available razor blades, tapes, tubes, and metal pins were reported as sufficient for cutting and to ensure attachment (Rhee and Somerville, 1995; Ayre and Turgeon, 2004; Nisar et al., 2012; Huang and Yu, 2015). In contrast to this technical simplicity, the accessibility of the graft junction or the developmental processes that can be analyzed is more limited compared to micrografting since tissues are older and in some cases, the junction not easily observed (Turnbull et al., 2002). When graft junctions form in mature tissues, there is less time for graft attachment and signal exchange than grafting in very young or juvenile tissues due to the short life span of *Arabidopsis*. Hence, there might also be limitations for phenotypic differences observed.

### Grafting of Rosettes

*Arabidopsis thaliana* forms a characteristic rosette that can be transplanted as well. Rosette grafting has been performed starting from the four rosette leaf stage onward and is suitable to study long distance signaling (Huang and Yu, 2015). Grafting in horticultural crop plants is often performed at a similar developmental stage in the stem region either above or below the first set of true leaves (Lee et al., 2010). Thus, rosette grafting in *A. thaliana* represents an analogous model to horticultural grafting in herbaceous species. The graft junction can be located in the rosette stem region (Figure 2J; Ayre and Turgeon, 2004; Chen et al., 2006; Huang and Yu, 2015), between the cotyledons and first true leaves in the epicotyl region (Figure 2E; Li et al., 2019) or below the cotyledons in the hypocotyl region (Figure 2I; Huang et al., 2012; Huang and Yu, 2015). Apart from shoot-root interactions, signaling within a shoot or between leaves can be studied by transplanting a scion with leaves and the apical meristem to a rootstock with leaves (Huang and Yu, 2015). For instance, signal molecule transport from one leaf to another could be studied more efficiently. Moreover, transgenerational inheritance studies could benefit from this technique since the graft junction forms before the inflorescence emerges and the reproductive tissue is formed. Limitations include preparation of appropriate grafting partners needs several weeks and also more space compared to micrografting. In addition, the visualization of the graft junction within the rosette is challenging due to the surrounding leaves and extremely short stem of *Arabidopsis* (Huang and Yu, 2015). Therefore, rosette grafting is not particularly suitable for studying graft junction formation.

### Grafting of Inflorescences

Inflorescence stem grafting (Figures 2K, 3H) was the first organ transplantation procedure established in *A. thaliana* (Tsukaya et al., 1993) and was then adapted in subsequent studies due to the highly accessible and relative ease to perform (Rhee and Somerville, 1995; Haywood et al., 2005; Huang and Yu, 2009; Lu et al., 2012; Nisar et al., 2012). Thus, studies of both graft junction formation and long distance signaling are possible. Moreover, high success rates of up to 87% were achieved and grafting can be done quickly (Nisar et al., 2012). The inflorescence stem of *A. thaliana* was previously used as a model system for wound healing and vascular regeneration (Asahina et al., 2011; Mazur et al., 2016) and future studies, including signals passing to developing seeds that allow transgenerational inheritance studies, will benefit from inflorescence stem grafting. Admittedly, inflorescence stem grafting requires more growth space and quite developmentally advanced plants than does rosette grafting. A possible horticultural relevance for inflorescence grafting is also lower than rosette grafting, since inflorescences are not the tissue that is usually used (Lee et al., 2010). As many developmental stages have already passed, the systemic movement of molecules across inflorescences might also be limiting compared to grafting at earlier stages (Turnbull et al., 2002).

## Transplanting Organs of *Arabidopsis thaliana* to Other Plant Species

So far, we have focused on intra-species grafting of *A. thaliana*. However, grafting within one species restricts the ability to investigate signaling pathways and why graft failure occurs since most self-grafts are successful in dicots. The more genetically similar two graft partners are, the less differences there are to identify mobile molecules. Furthermore, grafting in horticulture is most often done between different cultivars or closely related species to improve stress tolerance or modify growth phenotypes (Lee and Oda, 2010; Bie et al., 2017). To overcome these limitations and to provide greater relevance, grafting and generating chimeras between different species with an increasing evolutionary distance expands the range and depth of questions that can be addressed. For example, plant chimeras combining C_3_ photosynthesis of *A. thaliana* and C_4_ carbon fixation in *Cleome gynandra* (Newell et al., 2010) or salt tolerance in *E. salsugineum* and salt sensitivity in *A. thaliana* (Li et al., 2019) can be investigated by inter-species grafting. To this end, several protocols and reports of inter-species grafting were published during the past years. For instance, micrografting between scions and rootstocks of *A. thaliana* and the related *E. salsugineum* is possible (Amtmann, 2009; Melnyk et al., 2015; Li et al., 2019). A major issue of combining divergent species is to overcome graft incompatibility. *A. thaliana* can form graft junctions with members of the Brassicaceae family like *Brassica oleracea*, *Raphanus sativus* (Flaishman et al., 2008), *Capsella rubella*, *Cardamine hirsuta*, and *Olimarabidopsis pumila* (Melnyk et al., 2015), however, grafting of *A. thaliana* with more distantly related species is not possible in most cases. Inter-family grafts between *Nicotiana benthamiana* and *A. thaliana* seem to be exceptional (Notaguchi et al., 2012, 2015, 2020).

Different degrees of incompatibility are distinguished: short-term incompatibility is observable by failed vascular reconnection and wilted scions, whereas long-term incompatibility show vascular connections, but plant development or graft junction formation is affected later, in some instances months or years after the grafting event (Goldschmidt, 2014; Melnyk, 2017a,c). Additionally, other types of incompatibility need to be considered like that of grafted inflorescence scions of *A. thaliana* on stocks of *S. lycopersicum* which flower but their vasculature fails to connect (Flaishman et al., 2008). To solve this enigma, parasitic plants could serve as a model since they can connect their vasculature with evolutionarily distant plant species as their hosts through the formation of specialized invasive structures called haustorium. Interestingly, the parasitic plant *P. japonicum* not only forms natural root connections to various species, it can even be grafted to the distantly related *A. thaliana* (Kurotani et al., 2020). However, there is still limited information of which combinationcould serve as a good model for compatibility or incompatibility where the enhancement or inhibition of graft junction formation could be studied in detail. Nevertheless, inter-species grafting is gaining relevance in grafting research as it is of great agricultural importance and could be a useful tool to investigate scion-rootstock interactions (Warschefsky et al., 2016).

## Discussion

Sharrock’s book from 1660 illustrates the various types of grafting that are possible on a single tree, and nowadays, *A. thaliana* has become a modern Sharrock’s tree (Figure 1). A technique that began as a small part of a paper published in 1993 (Tsukaya et al., 1993) has now developed and emerged as a growing field of research used to understand graft formation, long distance signaling and rootstock-scion interactions. Grafting is possible in *Arabidopsis* hypocotyls, stems, inflorescences, roots, and leaves (Figure 2). With sufficient patience and skill, it is likely that most tissues of *Arabidopsis* can be grafted highlighting the remarkable developmental plasticity and wound healing abilities this plant has.

Various techniques have now been developed and the choice of which technique and tissue to use will depend on the experimenter’s needs, training and resources (Table 1). For many applications, hypocotyl (Figure 2A) or inflorescence grafting (Figure 2K) will be suitable and are the best tested and most widely used in the community. Intriguing grafting techniques include rosette grafting (Figures 2I,J), leaf grafting (Figure 2F) and root grafting (Figure 2H). These methods are not as widely used but could prove quite useful.

Grafting *Arabidopsis* has huge advantages – the speed, genetic resources, size of plants and their competency to rapidly graft both to themselves and to closely related species. The notable drawback for studying graft formation and rootstock-scion interactions is that there is seemingly little horticultural relevance for grafting Brassicaceae. However, it is likely that the ability to graft did not evolve independently in the Brassicaceae but instead shares common features with wound healing and vascular formation in many diverse plant species. As such, the developments and knowledge obtained in *Arabidopsis* should be tested in other relevant plant species and families. Discoveries made in *Arabidopsis* regarding graft formation have been confirmed in other species (Xie et al., 2019; Notaguchi et al., 2020), whereas discoveries made in *Arabidopsis* regarding long distance movement are now being tested in other plant species (Ye et al., 2014; Putterill and Varkonyi-Gasic, 2016; Zhao et al., 2020). Conversely, important findings made with grafting in non-*Arabidopsis* species such as organelle movement across the graft junction (Stegemann et al., 2012; Gurdon et al., 2016) and graft-induced dwarfing (Mudge et al., 2009) could be studied in *Arabidopsis* to better understand the mechanisms for these phenomena. It remains unknown whether enigmatic grafting processes like natural root grafting of trees can be studied in *Arabidopsis* analogously. Outstanding questions that deserve further attention include studying graft incompatibility using *Arabidopsis* and a suitable partner, developing robust protocols to graft organs such as roots and leaves, investigating the role of plasmodesmata and using divergent species grafts to better understand the range of molecules that move systemically. *Arabidopsis* will remain a powerful tool for grafting research and combined with horticulturally relevant species, will provide major insights into the processes of grafting, wound healing and vascular regeneration that should lead to an improvement in graft formation and a greater understanding of inter-tissue communication.

## Author Contributions

KB conceptualized the review. Both authors wrote the review, made figures, and edited and approved the final manuscript.

## Conflict of Interest

The authors declare that the research was conducted in the absence of any commercial or financial relationships that could be construed as a potential conflict of interest.
